# Comparison of TNF-α and IL-19 concentrations at different stages of breast cancer

**DOI:** 10.25122/jml-2021-0359

**Published:** 2022-06

**Authors:** Amera Kamal Mohammed

**Affiliations:** 1 Clinical Laboratory Sciences Department, College of Pharmacy, University of Kirkuk, Kirkuk, Iraq

**Keywords:** breast cancer, BMI, interleukin-19, TNF-α, metastasis

## Abstract

This study investigated the alteration of tumour necrosis factor (TNF-α) and interleukin-19 (IL-19) at different clinical disease stages, lymph node metastasis, and ductal carcinoma in women with breast cancer. Serum samples were collected from 90 individuals with an age range of 25–61 years. These individuals were divided into a control group (healthy people), consisting of 31 individuals, and a breast cancer patients (BCP) group, consisting of 59 individuals. The pathological data (tumour stage, lymph node metastasis, and ductal carcinoma) was obtained from the medical record of patients and confirmed by experienced histopathology. Enzyme-linked immunosorbent assay (ELISAs) technology was used to measure the serum concentrations of IL-19 and TNF-α. The results showed significant differences (P≤0.002) in the mean of BMI, interleukin-19, and TNF-α in BCP compared to controls, while the age factor did not play an important role. The stages of breast cancer caused clear differences in the levels of TNF-α and IL-19. According to the findings, BCPs had a greater level of TNF-α in lymph node metastases than healthy persons. The concentration of serum IL-19 in BCP with lymph node metastasis was significantly different in non-lymph node metastasis patients and healthy people. Additionally, BCP with ductal carcinoma showed significant differences in the mean levels of IL-19 and TNF-α in comparison with healthy people.

## INTRODUCTION

Breast cancer is the uncontrolled growth of breast cells that forms a malignant tumour, which could metastasize to other areas and tissues of the body [1]. Although this cancer primarily affects women, a few cases are reported in men [2, 3]. Breast cancer is one of the most frequent types of cancer globally, and it is the second leading cause of cancer-related deaths among women in the United States of America (40,610 deaths in 2017) [1]. Breast cancer is characterized by inflammation (such as immune cells) and some types of proteins (such as perforin), and pro-inflammatory cytokines [2]. It is noteworthy to mention that previous studies indicated that cytokines could motivate or inhibit the growth of breast cancer depending on two factors; the concentration of cytokines and the existence of modulating factors [3]. For example, Calcinotto et al. [4] reported that the presence of tumour necrosis factor (TNF-α) could promote the growth of breast cancer; this effect could be attributed to the ability of TNF-α to inhibit the immune response. At the same time, Calcinotto et al. [4] and Rao et al. [3] demonstrated that some types of cytokines, such as IL-6 and IL-18, could inhibit the progress of breast cancer by enhancing the anti-tumour ability of the immune system. Thus, the presence of TNF-α scarifying cells, such as macrophages, could promote the development of tumour cells [5]. Some researchers believe that the effect of TNF-α on the growth of breast cancer is not only due to its influence on the immune response but also its ability to induce the expression of angiogenic factors [6]. Although the mechanism of the influence of TNF-α on the development of breast cancer is not proven yet, a wide body of literature demonstrates that TNF-α plays a role in linking the inflammation and the growth of breast cancer [7].

Additionally, a high cancer risk has been found in individuals with elevated TNF-α levels [8, 9]. Other studies indicated that some interleukins, especially IL-19, act as mediators in breast cancer [8, 9], where high disease-specific survival (DSS) and metastasis-free survival (MFS) were noticed in patients with low levels of IL-19 compared with patients with high levels of IL-19 [10]. Moreover, Hsing et al. [10] indicated that IL-19 is also responsible for inducing fibronectin expression and cancer cell proliferation. It is noteworthy to highlight that anti-IL-19 monoclonal antibodies could be used to minimise the negative impacts of IL-19 [10]. In this context, the current study investigates the alteration of TNF-α and IL-19 at different clinical disease stages, lymph node metastasis, and ductal carcinoma in women with breast cancer.

Finally, it is noteworthy to mention that there are many potential reasons for the widespread cancer. For instance, some studies indicated that the consumption of polluted water with metals, nitrate, nitrite, by-products of chemical water disinfection, organics, phenols, or azo dyes could cause a wide range of cancers [11–14]. However, recent studies demonstrated that such pollutants could be removed efficiently from water using different treatment methods, such as adsorption, electrocoagulation and/or hybrid methods. In addition, air pollutants could also cause cancer [15]. Thus, it is recommended to use advanced technologies to monitor the concentrations of such pollutants in water and air.

## MATERIAL AND METHODS

The current study involved 90 individuals with ages ranging from 25–61 years; 59 people were diagnosed with breast cancer BCP (patients group), while the rest (31 people) were healthy (control group). The body mass index (BMI) of each participant has been calculated using the following equation [16]:


$$BMI=\frac{W}{H^{2}}$$

Where W and H represent the participant's weight (in kg) and height (in m), respectively, the pathological data (tumour stage, lymph node, metastasis, and ductal carcinoma) for each participant was obtained from the medical record of patients and confirmed by experienced histopathologist. In addition, serum concentrations of IL-19 and TNF-α were calculated by enzyme-linked immunosorbent assay (ELISAs).

## RESULTS

**Figure 1:**
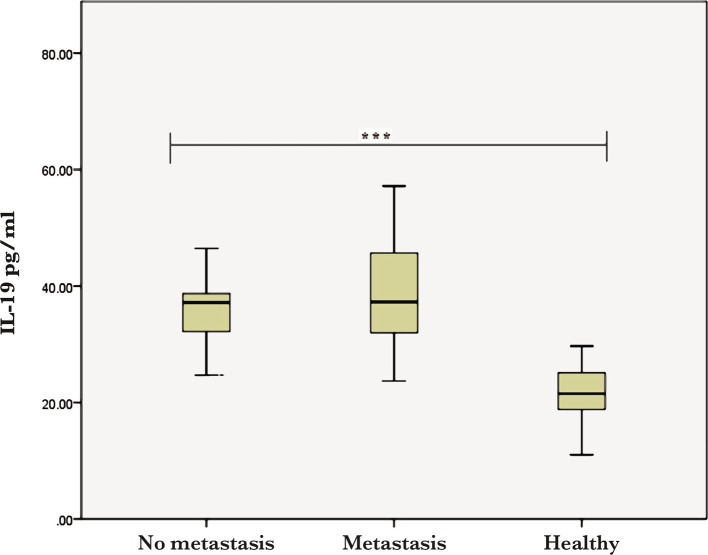
Serum IL-19 levels in healthy women and BCP with lymph node metastasis (*** – p<0.0001).

The results showed that there are significant differences (P≤0.002) between the BCP and controls in terms of BMI, IL-19, and TNF-α, while the age of participants did not show any significance (Table 1). Additionally, the levels of both TNF-α and IL-19 significantly varied (P<0.001) with the stage of the disease (Table 2). The obtained results also indicated a significantly increased level of TNF-α in the lymph node metastasis compared to healthy people (Figure 1).

**Table 1:** Comparison of age, BMI, TNF-α, and IL-19 according to the study groups.

Variable	Mean±SD Patient groups (n=59)	Mean±SD Control groups (n=31)	Significance (P)
**Age (year)**	37.39±8.39	7.70±34.74	0.12
**BMI (kg/m^2^)**	6.42±31.38	5.10±23.90	0.002*
**Serum TNF-α (pg/mL)**	7.43±30.89	2.86±12.50	0.001*
**Serum IL-19 (pg/ml)**	6.90±36.53	4.86±21.26	0.001*

BMI – Body Mass Index; TNF-α – Tumor necrosis factor-α; IL-19 – Interleukin 19; * – P-value is significant ≤0.05 levels; SD – Standard deviation.

**Table 2:** The differences between TNF-α and IL-19 at different stages of breast cancer.

Parameters		No.	Mean	Std. Deviation	LSD (0.05)
**TNF-α (pg/mL)**	**Stage I**	22	34.79	8.68	0.001*
	**Stage II**	17	29.66	5.19	
	**Stage III**	20	29.33	6.21	
**IL-19 (pg/mL)**	**Stage I**	22	36.17	7.52	0.008*
	**Stage II**	17	36.71	6.36	
	**Stage III**	20	39.51	11.99	

**Figure 2:**
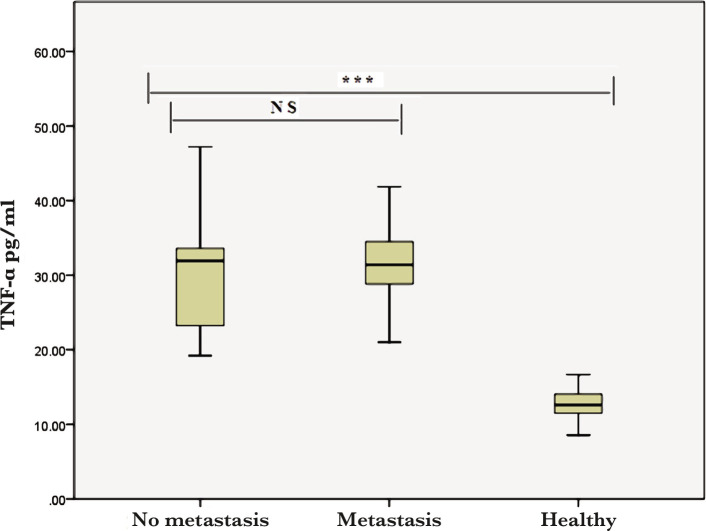
Serum TNF-α level in both healthy women and BCP with lymph node metastasis. NS – non-significant (*** – p<0.0001).

**Figure 3:**
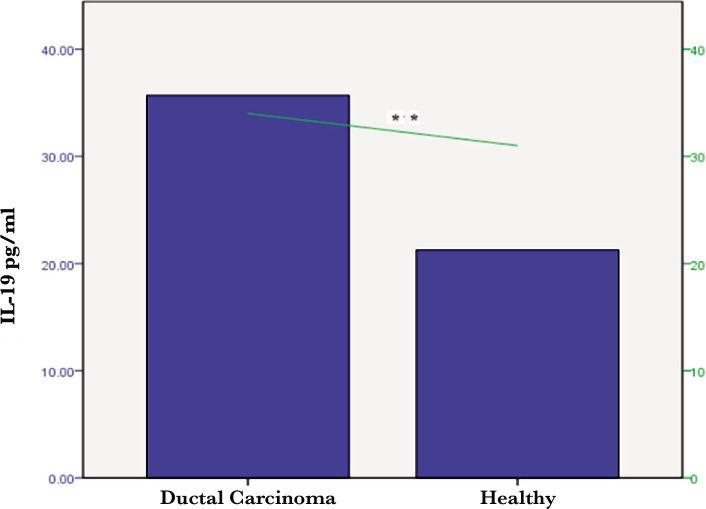
Serum IL-19 levels in healthy women and patients with ductal carcinoma (** – p<0.01).

**Figure 4:**
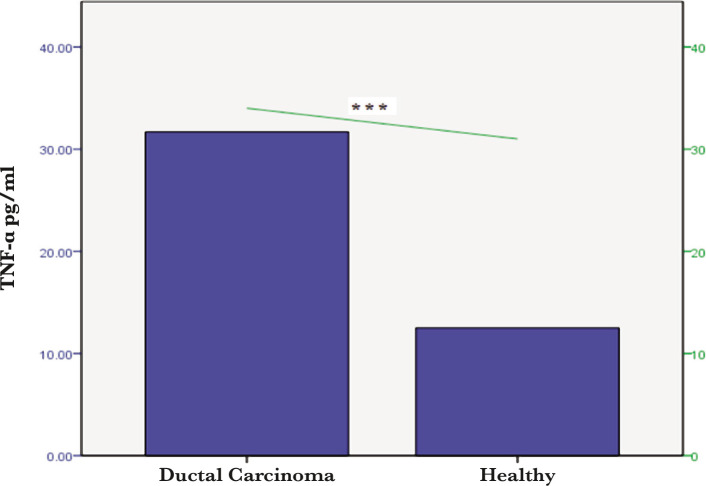
Serum TNF-α levels in healthy women and patients with ductal carcinoma (*** – p<0.0001).

However, there was no significant difference between the BCP and controls in terms of TNF-α in the lymph node metastasis compared to no lymph node metastasis (Figure 2). Individuals with ductal carcinoma recorded significant differences (p<0.001) in the mean levels of the interleukin-19 and TNF-α compared with healthy groups (Figures 3 and 4).

## DISCUSSION

It was found that the expression of the cell TNF-α in inflammatory breast carcinoma is related to the grade of the tumour. Additionally, it was found that the TNF-α expression is a key parameter in the metastatic behaviour of breast cancer. Furthermore, the results showed that the level of TNF-α was high in BCP with progressed tumour phenotypes in comparison with those with less progressed tumour phenotypes. Recently, some researchers believe that TNF-α plays a role in carcinogenesis by activating the nuclear factor kappa-light-chain-enhancer (NF-κB); the latter plays an important role in expressing cancer-related genes [17]. In addition, it was reported that TNF-α motivates the activation of the inducible nitric oxide synthase (iNOS), which is involved in different cellular changes resulting in malignancy [18]. Therefore, the results from the current investigation agree with previous studies [18, 19]. These findings could be explained by the fact that inflammation helps develop and progress cancer [9, 20].

In terms of IL-19, it was found that its expression is influenced by several factors, such as mitotic figures and the stage of the disease. Additionally, it was found that IL-19 directly helps proliferate and migrate breast cancer and indirectly promotes the progression of the tumour by providing the required microenvironment. A wide body of clinical and experimental works confirms that IL-19 is a key agent in breast cancer. It is involved in different processes, such as cell proliferation and migration, which accelerate tumour growth [21]. These results confirm that IL-19 is an indicator for breast cancer, which is in high agreement with the results of Chen et al. [21].

In terms of aging, the current study indicated no correlation between the age of participants and breast cancer. However, some previous studies indicated that other key factors, such as obesity and age, could motivate the tumour's genesis by providing the required inflammatory environment [22, 23]. Previous studies indicated three important facts. Firstly, the patients with breast cancer show apparent inflammatory responses [24]. Secondly, a direct correlation was found between the concentration of IL-19 and TNF-α and tumour grade in breast cancer patients [9]. Thirdly, IL-19 is an indicator for breast cancer as it helps to provide the required micro-environment for the progression of tumours [17, 25]. Finally, obesity contributes to tumorigenesis as it helps to develop an inflammatory environment [17, 26]. Thus, the literature recommends treating cancer by treating the inflammatory micro-environment [17, 27].

## CONCLUSIONS

Our results indicated that high levels of IL-19 and TNF-α, linked to clinical disease stage and lymph node metastasis, have an autocrine effect on breast cancer cells and produce a milieu conducive to tumour growth. Additionally, it could be concluded from the obtained results that inhibiting IL-19 could be helpful in the treatment of breast cancer.

For future studies, it is recommended to carry out the same research in other countries to check whether these results are applicable elsewhere too.
